# Impact of Reducing Obesity in PCOS: Methods and Treatment Outcomes

**DOI:** 10.3390/jpm15110518

**Published:** 2025-10-31

**Authors:** Alexa C. Dzienny, David B. Seifer

**Affiliations:** Department of Obstetrics, Gynecology, and Reproductive Sciences, Yale School of Medicine, New Haven, CT 06510, USA; david.seifer@yale.edu

**Keywords:** PCOS, obesity, dietary intake, physical activity, pharmacotherapy, personalized medicine

## Abstract

Obesity has become increasingly prevalent, impacting up to 41 percent of women in the United States between 2021 and 2023, leading to a rise in short- and long-term adverse health events. With regard to reproductive health, obesity is associated with menstrual irregularities, poorer reproductive and obstetric outcomes, and an increased risk of endometrial cancer. Obesity can lead to hyperandrogenism and anovulation, which is consistent with polycystic ovarian syndrome (PCOS). The prevalence of obesity is higher in women with PCOS compared to the general population. Although PCOS increases the risk of obesity, not all women with PCOS are obese, and not all women with obesity develop PCOS. However, individuals with both PCOS and obesity often present with a more extreme phenotype, with increased risk of chronic anovulation, glucose intolerance, dyslipidemia, metabolic syndrome, vitamin D deficiency, and decreased fertility. Therefore, weight loss is the backbone of patient management in women with obesity and PCOS, and is associated with improvement in cardiovascular risk, as well as improvement in menstrual cycles, ovulation, and pregnancy rate. Lifestyle modifications are often the first-line intervention, with data supporting low glycemic index diets, including ketogenic and DASH diets, along with vitamin D supplementation to improve hormonal imbalances, insulin sensitivity, and menstrual cycles in those who do not have normal vitamin D levels. Furthermore, with the recent widespread adoption of newer FDA-approved medications for weight loss, including GLP-1 (glucagon-like peptide) receptor agonists, new data are emerging regarding the impact of PCOS and longer-term cardiovascular risk. The treatment of PCOS requires a personalized approach, with consideration of a patient’s reproductive goals, tolerance of risk, and acceptance of behavioral and financial commitments, as well as consideration of other medical comorbidities. This narrative review explores different weight loss treatment options, comparing lifestyle modifications (including diet, physical activity, mindfulness, stress management, and cognitive behavioral training), weight loss medications, and bariatric surgery and their respective impact on PCOS to assist clinicians in guiding their patients towards an effective, individualized intervention.

## 1. Introduction

Polycystic ovary syndrome (PCOS) is the most common endocrinopathy impacting women globally, with prevalence ranging between 4 and 21% due to heterogeneity in diagnosis [1,2,3,4]. The Rotterdam Criteria were formally revised in the 2023 International Evidence-based Guideline for the Assessment and Management of Polycystic Ovary Syndrome [5]. To meet the criteria for the diagnosis of PCOS, patients must have two of the three clinical features—menstrual irregularity, clinical hyperandrogenism, and/or polycystic ovarian morphology (PCOM), as defined by elevated anti-Müllerian hormone (AMH) serum levels or ultrasounds [5]. When evaluating patients for PCOS, the 2023 International Evidence-based Guideline for the Assessment and Management of PCOS established a stepwise diagnostic algorithm. In this algorithm, irregular menstrual cycles and clinical signs or symptoms of hyperandrogenism in the absence of other causes for these manifestations are enough to make the diagnosis. Irregular menstrual cycles without clinical hyperandrogenism warrant biochemical testing for hyperandrogenism in the absence of other causes to rule in for diagnosis. If patients only present either irregular cycles or clinical or biochemical hyperandrogenism, then ultrasound or AMH testing for the evaluation of polycystic ovarian morphology can be used for diagnosis in an adult population in the absence of other causes [5]. With the various combinations of these features, PCOS can manifest as different phenotypes. The classic PCOS phenotype refers to hyperandrogenism, with ovulatory dysfunction with or without PCOM [3]. PCOS has significant reproductive implications, including menstrual irregularities, infertility, clinical hyperandrogenism with acne, hirsutism, female-patterned hair loss, and increased risk of endometrial cancer [5]. Furthermore, PCOS has greater implications for overall short- and long-term health, with an increased risk of dyslipidemia, type 2 diabetes, cardiovascular disease, metabolic syndrome, moderate to severe anxiety, depression, hypertension, and non-fatal cerebrovascular events [2,5,6,7].

**Figure 1:**
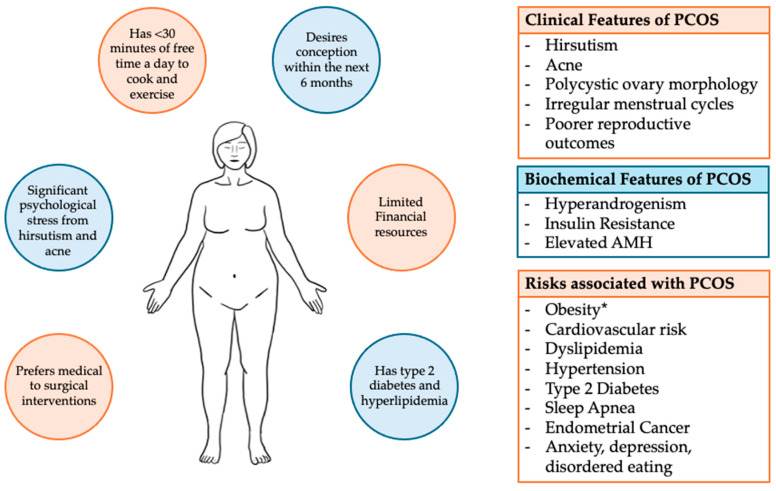
Clinical features that are potentially associated with PCOS, and examples of those personal priorities and preferences that guide care. * Obesity is present in approximately 30 to 75% of patients with PCOS, with estimates varying widely depending on specific populations and geo-ethnic groups. Obesity and PCOS will together synergistically increase cardiometabolic risk.

Approximately 30 to 75% of patients with PCOS are obese, although these estimates vary widely depending on specific populations and ethnic groups [6]. PCOS serves as a risk factor for obesity. Obesity can, similarly and independently of PCOS, increase cardiovascular and metabolic risk and is associated with poorer reproductive outcomes, including high rates of miscarriages, increased obstetric complications, longer time to conception, and lower fertility rates [1,6,8,9]. When presenting together, the combination of obesity and PCOS acts synergistically to exacerbate insulin resistance, hyperandrogenism, and cardiovascular-related comorbidities [1,10]. The mainstay of first-line treatment for both PCOS and obesity involves weight loss and lifestyle modifications. Currently, there is neither a specific diet, exercise regimen, nor behavioral intervention that is universally recommended to achieve the goal of maintaining a healthy weight [5]. With minimally invasive bariatric surgeries becoming widely adopted alongside the rapid acceptance of newer anti-obesity medications, including GLP-1 (glucagon-like peptide) receptor agonists, there are now more viable options conferring different risks and benefits to help guide patients to achieve their goals. However, given the variability in phenotypes, comorbidities, both short-term and long-term health risks, and patients’ own goals and reproductive plans, a personalized approach is required to best support and care for patients in a whole-body, holistic approach. Figure 1 depicts the variations in clinical presentation, associated biochemical markers, and health risks of PCOS alongside competing patient preferences, goals, and priorities that need to be considered when approaching management options. In this narrative review, we provide a comprehensive overview of the intersection of PCOS and obesity and the available evidence regarding different treatment options to assist clinicians in developing personalized interventions for their obese patients with PCOS.

## 2. Pathophysiology of PCOS

PCOS is characterized by hyperandrogenism, menstrual and ovulatory dysfunction, and polycystic ovarian morphology. PCOS is also associated with an increased risk of insulin resistance independent of weight and BMI (body mass index), impacting 50 to 70% of patients with PCOS [6]. Insulin resistance contributes to PCOS, serving as an independent risk factor for metabolic dysfunction [6]. Approximately 20–30% of women with PCOS meet the diagnostic criteria for metabolic syndrome [3,9]. Metabolic syndrome is a constellation of conditions that together increase the risk of cardiovascular disease, requiring at least three of the following for diagnosis: abdominal obesity, high blood pressure, elevated fasting glucose, elevated triglycerides, or reduced HDL (high-density lipoprotein) cholesterol. Patients with PCOS are also at increased risk of the development of type 2 diabetes [3,5,6,7].

Insulin resistance and hyperandrogenism are hallmarks of classic PCOS and function synergistically to increase metabolic and cardiovascular risk [6]. Hyperandrogenism in PCOS has been correlated with an increased frequency of gonadotropin-releasing hormone secretion, increased serum luteinizing hormone levels, excessive ovarian theca cell androgen production, and elevated serum AMH [10]. Theca cells in the ovary have increased insulin sensitivity, even at physiological insulin levels [8,10]. Therefore, in a state of insulin resistance with higher circulating levels of insulin, theca cells are stimulated to produce excess androgens [2,6]. In women with PCOS, cultured luteinized granulosa cells also exhibit a stimulatory response to insulin’s mitogenic actions, promoting androgen production [3,11]. The local accumulation of follicular androgens then promotes premature follicular atresia, contributing to anovulation [6]. Insulin also decreases sex hormone-binding globulin production, which increases the free bioavailable testosterone [10].

AMH has been hypothesized to be a biochemical mediator, contributing to polycystic ovarian morphology and hyperandrogenism [12]. AMH is a homo-dimeric glycoprotein hormone that is part of transforming growth factor-β, which is produced as a pro-hormone that is cleaved after secretion with a covalently linked biologically active C-terminal fragment [12]. In females, AMH is exclusively expressed by granulosa cells, with peak concentration at FSH-dependent preantral and small antral follicles of <4 mm with declining levels with follicular growth allowing the development of a dominant follicle [12]. As previously noted, AMH has been found to be markedly elevated in patients with PCOS. PCOM is characterized by a higher number of preantral and smaller antral follicles, with arrested development at the peak expression of AMH [12]. Furthermore, AMH has been found to be positively correlated with small antral follicle count and negatively correlated with serum FSH concentration, playing a role in early follicular arrest [12]. Elevated AMH has been associated with slowing FSH-sensitive follicular growth and with a lower apoptosis rate of granulosa cells from preantral follicles [6]. The concentration of AMH in follicular fluid has been found to be fivefold greater in anovulatory women compared with ovulatory women, strongly suggesting that AMH plays a role in ovulatory dysfunction and the development of PCOM [12,13]. In both mouse and human cells, AMH has been associated with the downregulation of aromatase in granulosa cells, increasing the synthesis of androgens within the follicle and exacerbating the hyperandrogenic micro-environment [12]. Given the well-described association between AMH as a surrogate marker for PCOM in a meta-analysis, with a pooled sensitivity of 0.79 and specificity of 0.87, serum AMH assays have been adopted as an alternative to ultrasound for the diagnosis of PCOM in terms of the diagnostic criteria for PCOS [5,14]. This change in the practice guidelines provides an easier, more cost-effective method for diagnosing PCOS by alleviating the barriers associated with ultrasounds, including scheduling, sonographer expertise, and imaging limitations with transvaginal or transabdominal approaches [15].

### 2.1. Role of Vitamin D Deficiency

Vitamin D deficiency has also been extensively reviewed as a potential pathway for contributing to the presentation of PCOS. Vitamin D deficiency has been found to correlate with PCOS, with an estimated 65 to 85% of women with PCOS having concurrent vitamin D deficiency [6]. Vitamin D deficiency is associated with decreased levels of SHBG (sex hormone-binding globulin), resulting in greater circulating levels of free testosterone, thereby contributing to hyperandrogenism [3]. Furthermore, Vitamin D has been found to have an inverse relationship with BMI and waist circumference in the PCOS population, indicating a potential relationship between obesity, PCOS, and vitamin D insufficiency [6]. Women undergoing ovulation induction for infertility and who have vitamin D deficiency have poorer reproductive outcomes than those with PCOS who have normal vitamin D levels [16]. In animal models, vitamin D deficiency has been associated with impaired folliculogenesis and steroidogenesis [6]. Furthermore, in the PCOS population, vitamin D has been found to have an inverse relationship with indices for insulin resistance, including HOMA-IR (the homeostatic model assessment for insulin resistance), with this relationship being more pronounced in sub-group analysis with individuals with elevated BMI [16]. Similarly, Vitamin D has been shown to have a positive correlation with SHBG levels [3]. However, the pathway of this relationship is unclear, due to the confounding factor of insulin resistance, which has also been shown to have an inverse correlation with both Vitamin D and SHBG levels [6].

One hypothesis for the pathway that is involved in Vitamin D’s role in the phenotype of PCOS concerns the dysregulation of TGF-β (transforming growth factor beta). Patients with PCOS have been found to have increased levels of circulating free TGF-β and decreased concentrations of soluble endoglin (sENG), which binds TGF-β1 and decreases its bioavailability [17]. In other fibrotic disease states, vitamin D was found to decrease TGF-β1. An RCT (randomized control trial) was performed to determine the effect of vitamin D supplementation on individuals with PCOS and Vitamin D deficiency on TGF-β1. The study found that vitamin D supplementation reduced TGF-β1 bioavailability and increased serum sENG values. Additionally, the study confirmed the positive impact of supplementation on clinical hyperandrogenism, triglycerides, and menstrual cycles [18]. There is also evidence supporting the role of VEGF (vascular endothelial growth factor) overexpression in women with PCOS and of potential angiogenesis dysregulation also contributing to the diagnosis of PCOS and the associated poor reproductive outcomes [19]. While these studies are promising, the exact role of Vitamin D and its pathophysiological pathways in terms of contributing to the PCOS phenotype still requires further human cellular examination and further randomized controlled trials to determine the cause-and-effect relationships between these variables.

### 2.2. Role of Chronic Inflammation and the Gut Microbiome

In addition to Vitamin D deficiency, understanding the interplay between states of chronic inflammation and the gut microbiome with PCOS, insulin resistance, and hyperandrogenism is an area of active research. In a meta-analysis reviewing circulating inflammatory markers in PCOS, C-reactive protein (CRP) was found to be the most reliable inflammatory marker in this population [20]. CRP levels were found to be 96% higher in individuals with PCOS compared to controls, independent of BMI [20]. Individuals with PCOS and a normal weight have also been found to have higher circulating levels of TNF-α (tumor necrosis factor-alpha) compared to healthy controls, supporting evidence that PCOS is a pro-inflammatory state, even in the absence of obesity [21]. PCOS is associated with circulating mononuclear cells (MNC) that, with the consumption of glucose and saturated fat, induce a pro-inflammatory response [6]. Glycolysis and the beta-oxidation of lipids within MNCs result in downstream effects promoting the secretion of reactive oxygen species (ROS), TNF-α, and IL-6 (interleukin 6) [6]. In both rat and human models, TNF-α stimulates theca cell proliferation, supporting the role of local inflammation contributing to hyperandrogenism [22]. Furthermore, studies have found that antioxidant therapy with resveratrol and statins suppresses local inflammation, and supplementation is associated with lower ovarian and adrenal androgen production [23,24,25].

The gut microbiome has emerged as playing a role in understanding metabolic dysfunction, inflammation, and insulin resistance. The gut microbiome has been associated with the pro-inflammatory state and insulin resistance in other metabolic disorders, including obesity [26]. The gut microbiome is analyzed both in abundance and in the diversity of species in one environment, alpha diversity, and as a community ecosystem between different environments, beta diversity [26]. Individuals with PCOS have been found to have reduced alpha diversity compared with healthy controls in terms of species richness. Measures of overall species diversity negatively correlated total testosterone levels with hirsutism in individuals with PCOS [27]. Individuals with PCOS have also been found to show a hierarchical clustering of species that can distinguish samples from healthy controls [27,28]. Specifically, the microbiome of individuals with PCOS exhibits a decrease in the species of bacteria responsible for producing short-chain fatty acid- and bile acid-metabolizing bacteria, contributing to a pro-inflammatory environment [29]. Short-chain fatty acids are produced by gut bacteria metabolism and have an anti-inflammatory effect, and contribute to maintaining gut barrier function [26,29]. Short-chain fatty acids have been suggested to participate in glucose-stimulated insulin secretion, improving insulin sensitivity and the secretion of glucagon-like peptide and peptide YY [26,30]. Therefore, a decrease in short-chain fatty acids has been hypothesized as a mechanism exacerbating insulin resistance in individuals with PCOS. Furthermore, the microbiome of individuals with PCOS shows increased representation of pro-inflammatory bacteria such as Fusobacterium, a bacterium associated with promoting inflammation in other metabolic disorders [29].

## 3. Relationship Between Obesity and PCOS

Based on recent data from 2021–2023, approximately 40.3% of adults in the United States are obese, with an increase in the prevalence of severe obesity compared to 2013–2014 data [31]. Based on data from the World Health Organization (WHO), in 2022, 2.5 billion adults over the age of 18 were overweight, approximately 43% of them, with significant geographic variations [32]. Based on the CDC (Centers for Disease Control) and WHO criteria, obesity is defined as a BMI value greater than or equal to 30 kg/m^2^ and severe obesity as a BMI value over 40 kg/m^2^, although this review recognizes the limitations of BMI values in accounting for different body types and fat versus muscle distribution, ethnic variations in metabolic risk based on BMI, and lack of distinction for central adiposity. Obesity is an independent risk factor for poorer obstetric outcomes, including a longer time to conception, lower fertility rates, an increased need for gonadotropins, and higher rates of miscarriages [6,33]. Obesity has been found to be more prevalent in individuals with amenorrhea compared to individuals with normal menstrual cycles [34,35]. Both BMI and central adiposity have been associated with reduced odds of ovulation in response to clomiphene in anovulatory women [34]. Furthermore, obesity has been associated with increases in duration and amount of gonadotropins for oocyte stimulation and retrieval, while also being associated with reduced oocyte yield and a greater risk of cycle cancellation [34]. In addition to the reduced quantity of oocytes retrieved, obesity is also a risk factor for exhibiting a poorer quality of oocytes [34]. This is proposed to be due to an altered follicular microenvironment exhibiting oxidative stress, inflammation, and insulin [34]. In mouse models, obesity was associated with mitochondrial dysfunction in oocytes, resulting in oxidative stress and impaired meiosis, along with irreversible changes despite weight loss [36,37]. Compared to women without obesity, women with obesity experience a greater percentage of euploid spontaneous abortion when the products of conception are evaluated, with higher BMI being a significant risk factor for miscarriage [34]. In terms of pregnancy, obesity confers increased obstetric risk, including gestational diabetes, pregnancy-related hypertension and preeclampsia, and cesarean sections. Obesity is also associated with generally poorer health outcomes, including increased cardiovascular risk, metabolic syndrome, dyslipidemia, hypertension, obstructive sleep apnea, and cancer [10].

Up to 75% of patients with PCOS are also obese, and PCOS is a risk factor for developing obesity [6]. Women with obesity can also develop insulin resistance and hyperandrogenism, which exacerbates the phenotype of PCOS when the two conditions are comorbid [1,10]. A small prospective study compared four groups of patients, based on BMI and PCOS diagnosis, who were undergoing IVF (in vitro fertilization) to evaluate differences in hormonal profiles and IVF outcomes. Patients with normal BMI and overweight BMI values without PCOS were compared to patients with PCOS in the normal and overweight BMI categories [38]. This study found that patients with PCOS had elevated LH levels and AMH compared with controls [38]. However, patients with PCOS and an overweight BMI were found to have significantly elevated total testosterone and androstenedione levels compared to all study groups [38]. Both subgroups including patients with obese BMI values had elevated HOMA-IR levels independent of PCOS. However, while the overweight control group did not have a significantly higher HOMA-IR compared to the normal weight control group, the subgroup with obesity and PCOS had a significantly increased HOMA-IR compared to the control normal-weight group, indicating the synergistic role of both PCOS and obesity in insulin resistance [38]. White adipocytes in excess adipose tissue express a cytochrome P450-dependent aromatase that is responsible for converting androgens to estrogen within fat [6]. Other adipocytes express 17β hydroxysteroid dehydrogenase, which converts androstenedione to testosterone and estrone to estradiol, which are more potent androgens and estrogens, respectively. Furthermore, individuals with PCOS who are also obese are more likely to experience chronic anovulation [6,10]. Increased unopposed estrogen levels increase the risk of endometrial hyperplasia, with progression to endometrial adenocarcinoma, in women with obesity [1]. Women with obesity have also been found to have lower levels of SHBG in comparison to normal-weight women with PCOS, contributing to increased circulating levels of free testosterone [9]. Obesity has also been associated with hyperstimulation of the hypothalamic–pituitary–adrenal axis, contributing to increased adrenal androgen secretion and increased LH levels [6,10]. Therefore, obesity can contribute to and worsen the hyperandrogenic state that is present with PCOS.

While insulin resistance and cardiometabolic risk are elevated in patients with PCOS regardless of BMI, the degree of insulin resistance is more profound in individuals with co-morbid PCOS and obesity compared to their normal-BMI counterparts. Insulin resistance in lean individuals with PCOS affects their postprandial glucose levels. However, in individuals with PCOS and obesity, insulin resistance impacts fasting glucose levels as well [6,39]. Alterations to cytokines and inflammatory markers have also been implicated with obesity and PCOS, including decreased adipokine levels and increased TNF-α and IL-6, contributing to increased steroidogenesis, hyperandrogenemia, and insulin resistance [10]. In addition to insulin resistance, there is an increased prevalence of dyslipidemia and metabolic syndrome in individuals with co-morbid obesity and PCOS [1,10,40].

The synergistic impact of obesity and PCOS translates into more significant clinical manifestations, including greater reports of hirsutism, anovulation, infertility, cardiometabolic risk, and impaired psychological well-being [1].

## 4. Weight Loss and PCOS

Given the additive reproductive, metabolic, and cardiovascular risks with the co-existence of PCOS and obesity, the first-line intervention in these patients is weight loss and lifestyle modifications with the goal of losing at least 5% of body weight, which has been associated with improved ovulation, hyperandrogenism, and insulin sensitization [6,9]. Weight loss has also been associated with improved metabolic parameters, including lower fasting blood glucose, fasting insulin, lipid profile, blood pressure, and free testosterone levels [1]. Furthermore, weight loss was associated with improved psychological health and better menstrual regularity [1,41,42].

Weight loss has also been associated with improved reproductive outcomes, with a 10% reduction in body weight being associated with an improvement in live birth rate in overweight women seeking treatment for infertility, as reported in a retrospective cohort study of 32 patients [43]. However, in randomized controlled trials, there has been insufficient evidence to confirm the beneficial effect of weight loss on live birth rate, although weight loss has been associated with increased rates of unassisted conception [5,44,45]. In a Danish National British Cohort study of patients who were overweight or obese, weight loss was associated with fewer days to conception, with 1 kg weight loss on average corresponding to 5.5 fewer days [33]. Given conflicting data regarding weight loss’s impact on fertility outcomes, it is important to assess the patient’s goals and their additional medical comorbidities when developing a treatment plan. For example, in a randomized trial of patients with a BMI of 29 kg/m^2^ or higher and infertility, patients were randomly assigned to groups that either proceeded to infertility treatment or participated in a 6-month lifestyle intervention prior to infertility treatment. In this study, the lifestyle intervention group for obese infertile patients did not result in improved live birth rates once infertility treatment was initiated [45]. Furthermore, based on a retrospective cohort study using SART CORS (Society for Assisted Reproductive Technology Clinic Outcome Reporting System) data on IVF cycles, which evaluated the impact of age and BMI on cumulative live birth rate, they found that in older ages, an age-related decline in fertility had a larger impact on the cumulative live birth rate than BMI [46]. Therefore, it is prudent to assess the patient’s overall reproductive and competing health goals along with their characteristics to curate an individualized treatment plan [46].

In addition, AMH has been found to be a potential predictive biomarker for the clinical response to weight loss in patients with PCOS [12]. Patients with a lower baseline AMH prior to weight loss were found to experience greater improvements in menstrual cyclicity and ovulatory function following weight loss, compared to patients with a higher baseline AMH [47], although weight loss did not decrease the AMH concentrations [47]. Further studies will be required to validate this association; however, AMH may serve to identify potential poor responders to weight loss, allowing for the earlier initiation of medical treatment [47].

## 5. Obesity Management and PCOS Outcomes

### 5.1. Lifestyle Changes

#### 5.1.1. Diet

Based on the International Evidence-based Guideline for Assessment and Management of PCOS, currently, there is no one specific type of diet composition that is recommended; however, a healthy, sustainable diet tailored to a patient’s own preferences is advised [5].

##### Low Glycemic Index

As PCOS and obesity both have an increased risk of insulin resistance, low glycemic index diets have been studied as a way to improve outcomes. A low glycemic index (GI) diet is defined as foods yielding a slow, steady rise in blood sugar levels (i.e., whole grains, fruits, and vegetables) to prevent rapid spikes in serum insulin and glucose levels [2]. Low glycemic index diets have been associated with anti-inflammatory properties, along with increased uric acid and glutathione peroxidase activity [2]. The low GI diet has been associated with improved ovulatory cycles and menstrual regularity compared to a standard healthy macronutrient-matched diet [48]. Even with limited weight loss of 4–5% of body weight, a low GI diet was associated with a threefold improvement in whole-body insulin sensitivity, which is measured by the insulin sensitivity index following an oral glucose tolerance test in patients with PCOS [49,50]. In a short 16-day trial of a low glycemic diet compared with a monounsaturated fatty acid-enriched diet for individuals with PCOS, the low GI diet was associated with decreased fasting insulin and total cholesterol levels compared with a high GI, low-fat diet [50]. In a meta-analysis comparing different diets for the management of PCOS, a low GI diet was associated with significantly reduced waist circumference, HOMA-IR, fasting glucose, and BMI compared to minimal intervention [51].

##### Ketogenic Diets

Ketogenic diets (KD) have a low glycemic index and have become increasingly popular due to their advantageous impact on metabolic biomarkers in patients with type 2 diabetes. Ketogenic diets have been associated with significant weight loss, decreased BMI, total cholesterol, and triglycerides with improved insulin resistance [2]. The ketogenic diet is characterized by a low-carbohydrate diet with adequate protein and a low to high fat content [2,52]. The lower carbohydrate content and increased fat content promote the hepatic production of ketone bodies as an energy source, thereby leading to ketosis, which functions to help suppress appetite [52]. Beyond a classic ketogenic diet that is high in fat, there has been further research studying very-low-calorie ketogenic diets (VLCKD) or very-low-energy ketogenic therapy (VLEKT), which is characterized by a more restrictive calorie intake and lower fat content [52]. In patients with obesity, VLCKD or VLEKT has been associated with significant weight loss in both the short and the long term, with improved metabolic parameters, including lower hemoglobin A1c and LDL (low density lipoprotein), as well as anthropomorphic parameters, including fat mass and waist circumference [52,53]. However, given a restrictive caloric intake, patients require medical supervision and a stepwise plan with regular laboratory monitoring for electrolyte imbalances, dehydration, and nutritional deficiencies. Therefore, this specific diet may serve a specific subset of individuals with severe obesity who would benefit from rapid weight loss and who are able to undergo significant lifestyle changes and frequent medical monitoring, which may not be an option for all individuals in terms of the accessibility of resources, coverage, and time availability to commit to the regimen.

Specifically regarding PCOS, ketogenic diets have been reported to lower androgen levels, decrease the LH to FSH ratio, and increase sex hormone-binding globulin. The hypothesized mechanism is due to reduced excess insulin production, with lower circulating glucose promoting a lipolytic effect with the loss of fat mass, which contributes to decreased peripheral aromatization and the production of excess androgens [2,3]. A recent systematic review and meta-analysis studied the impact of VLEKT compared to traditional high-fat ketogenic diets, specifically in patients with comorbid PCOS and increased body weight. In this study, when individuals compared their former diets prior to intervention with either VLEKT or high-fat KD, both VLEKT and high-fat KD regimens led to improvements in weight, BMI, and fat and lean mass percentages, in addition to lower serum glucose, HOMA IR, cholesterol, and LH and total testosterone levels. Similarly, both forms of ketogenic diets led to a reduction in serum glucose and HOMA IR scores in comparison to standard low-calorie diets. However, VLEKT was associated with a greater reduction in fat mass, mild superiority in terms of an improvement in glycometabolic profile, and lower triglyceride levels compared to high-fat ketogenic diets, indicating a potential further benefit from VLEKT, although both ketogenic diets generally were superior to the patients’ former diets or low-calorie diets [52].

##### DASH Diet

The Dietary Approaches to Stop Hypertension (DASH) diet was developed for the management of hypertension [54]. Since its inception, the DASH diet has now been found to also have benefits in terms of lowering blood glucose levels, cholesterol, and insulin resistance and has been adopted as a tool for managing type 2 diabetes and heart failure, with some studies indicating its role in reducing all-cause mortality in adults [54]. The DASH diet is a balanced diet emphasizing the consumption of vegetables and fruits, lean meats, and low-fat dairy, including micronutrients (potassium, calcium, and magnesium), with sodium restricted to 1500 mg/day [54]. The DASH diet emphasizes “healthy” carbohydrates to include micronutrients with a low glycemic index [54]. The DASH diet has also been found to have benefits for reproductive health [2]. In a meta-analysis of women with PCOS comparing diets on insulin resistance, the DASH diet was found to significantly improve HOMA-IR, fasting insulin, fasting glucose, BMI, and weight [51]. The DASH diet outperformed low-carbohydrate diets in terms of HOMA-IR score, although it was as effective as a low-carbohydrate diet for fasting insulin levels [51]. A subsequent meta-analysis comparing dietary interventions in the management of PCOS also confirmed that the DASH diet, when compared to a control normal diet, had the most superior effect in reducing HOMA-IR compared to low-carbohydrate, low-calorie, and Mediterranean diets [55]. This is consistent with research regarding the DASH diet for the management of type 2 diabetes. However, the DASH diet was not found to significantly reduce total testosterone levels in women with PCOS [55].

In a recent cross-over, randomized control study that was performed in the United Kingdom, the impact of a diet rich in ultra-processed food versus minimally processed food on weight loss and other anthropometric parameters was studied when following the United Kingdom’s Eatwell Guide regarding a healthy diet [56]. This study revealed greater weight loss, BMI reduction, and fat mass loss with minimally processed foods compared with ultra-processed foods when following a “healthy diet” along with guidance on the composition of macronutrients and food groups [56]. While this population did not include patients with PCOS, this study emphasizes the importance of the source of the ingredients as well as general macronutrients when approaching a diet. However, further randomized trials would be necessary to clarify the impact of ultra-processed foods on improving the PCOS phenotype.

In summary, there are dietary interventions that can improve both metabolic parameters and hormonal imbalances in women with PCOS. In selecting appropriate data, a personalized approach is preferred to tailor the intervention to support patients based on their lifestyle availability and ability to commit to the diet, other comorbidities, and their phenotype of presentation. For example, low-calorie diets have been found to be more effective for rapid weight loss; however, the DASH diet or low glycemic index foods may be better for specifically targeting insulin resistance. Furthermore, patient preference in terms of food selection is required to ensure sustainable changes.

#### 5.1.2. Dietary Supplementation

In addition to changing the overall macronutrient composition of diets, several dietary adjuncts and supplementations have been proposed to help improve the downstream effects of PCOS and obesity by addressing the underlying pathophysiology of disease states. For example, given the correlation between vitamin D deficiency and biochemical changes and the clinical features of PCOS and obesity, supplementation has been reviewed in this population. Furthermore, supplements have been reviewed to address the underlying role of the gut microbiome and low-grade chronic inflammation and oxidative stress on the sequelae of PCOS and obesity.

##### Vitamin D Supplementation

Given the association of vitamin D deficiency with PCOS and obesity, multiple studies have evaluated the role of vitamin D supplementation. In a single-arm open-label trial of women with PCOS who were overweight and had vitamin D deficiency, a 12-week supplementation of vitamin D was associated with improved 25-hydroxy-vitamin D levels, decreased total testosterone and androstenedione levels compared to baseline, and lower blood pressure parameters in patients with elevated blood pressure at baseline. However, in this study, the HOMA IR was unchanged [57]. In a double-blind, placebo controlled trial on patients with PCOS and vitamin D deficiency, vitamin D supplementation was found to improve glucose homeostasis, CRP (C-reactive protein) levels, and malondialdehyde (MDA)^2^. In a meta-analysis, a 12-week supplementation of vitamin D in patients with PCOS similarly improved total testosterone levels, total antioxidant capacity, and MDA and CRP without a significant effect on SHBG, free testosterone, and DHEA-S [58]. Given the association of PCOS with elevated AMH, vitamin D supplementation was also associated with a decrease in serum AMH values and an increase in the soluble receptor for advanced glycation end products, a protein thought to mitigate the effects of circulating products of oxidation [12]. Based on current data, vitamin D supplementation could be a reasonable and low-risk option for a population of women with PCOS and vitamin D deficiency, especially in those with an elevated BMI.

##### Antioxidant Supplementation

As both obesity and PCOS have been studied for their association with oxidative stress and pro-inflammatory states, both anti-inflammatory and anti-oxidative diets have been studied. An anti-inflammatory diet rich in fruits, vegetables, omega-3 fatty acids, and whole grains has been associated with an improvement in circulating pro-inflammatory markers, including IL-1 (interleukin 1), IL-6, and TNF-alpha [2]. Specifically, omega-3 fatty acids have been found to support healthy cardiovascular and nervous systems, mood, the skin, and immune function [2]. Foods high in omega-3 fats include cold-water fish, seeds, green leafy vegetables, nuts, and beans. Omega-3 fatty acids, such as alpha-linolenic acid (ALA), eicosapentaenoic acid (EPA), and docosahexaenoic acid (DHA), have been associated with decreases in cholesterol and triglycerides and improved insulin sensitivity [6]. In a randomized controlled trial involving women with PCOS who were overweight or obese, omega-3 fatty acid supplementation was associated with an improvement in serum adiponectin, insulin resistance, and lipid levels [59]. Other studies have similarly shown improvements in LH, a decreased LH to FSH ratio, and improved menstrual cycles [2,60,61].

For antioxidants, both coenzyme Q10 and vitamin E supplementation have been studied. In a double-blinded placebo-controlled randomized control study in women of reproductive age with PCOS, comparing 100 mg of daily CoQ10 supplementation to a placebo for 12 weeks, coenzyme Q10 was associated with decreased CRP, total testosterone, DHEA-S, hirsutism, MDA, and total antioxidant capacity [62]. Antioxidant therapy is responsible for stabilizing cell membranes and preventing mitochondrial dysfunction by preventing the accumulation of reactive oxygen species [3]. In a meta-analysis reviewing vitamin E supplementation in women with PCOS, vitamin E was associated with reduced fasting glucose and insulin levels, HOMA-IR, total cholesterol, triglycerides, and total testosterone and an increase in SHBG without a significant difference in HLD, BMI, and hirsutism. Vitamin E is thought to up-regulate PPAR-γ (peroxisome proliferator-activated receptor gamma), which subsequently up-regulates adiponectin [63]. Given the promising early results of animal studies showing resveratrol having a beneficial impact on suppressing theca cell proliferation, resveratrol has been studied as a potential therapeutic supplement for individuals with PCOS. In a systematic review of resveratrol and PCOS, in animal studies, resveratrol improved polycystic ovarian morphology and estrus cyclicity, reduced serum testosterone and LH, and reduced pro-inflammatory markers, including TNF-α, IL-6, and MAD levels in serum and ovarian tissue [64]. However, there was mixed evidence regarding the effect of resveratrol supplementation on glycemic control and lipid profile in animal studies [64]. In a subsequent systematic review and meta-analysis of resveratrol supplementation in PCOS, resveratrol was associated with improved prolactin levels, acne, and total cholesterol, without significant improvements in the hormonal profile, BMI, and individual lipids [65].

##### Probiotic Supplementation

Given the evidence of gut dysbiosis in individuals with PCOS contributing to insulin resistance and hyperandrogenism, probiotic supplementation as a potential therapeutic option has been studied. Probiotic supplementation contains live organisms, while prebiotic supplementation refers to nondigestible substances that contribute to the growth of microbes, and synbiotics combine both types to improve the survival of microbes. In a meta-analysis of seven trials studying probiotic supplementation in women with PCOS, there was a significant reduction in triglyceride levels, fasting insulin, and HDL without a significant impact on HOMA-IR, fasting blood glucose, LDL, total cholesterol, CRP, and anthropometric indices (i.e., weight, BMI, and waist circumference) [66]. However, another meta-analysis of eight trials studying probiotic supplementation in women with PCOS did find a significant improvement in mean differences in weight or BMI, fasting blood sugar, insulin, and HOMA-IR levels, as well as in total testosterone in this population [67]. In a systematic review comparing probiotic, symbiotic, and prebiotic supplementation in women with PCOS, symbiotic supplementation was associated with the greatest reduction in HOMA-IR, fasting blood sugar, and insulin levels, followed by probiotic supplementation and, lastly, prebiotic supplementation, though all supplementation was associated with benefits in terms of insulin sensitivity [68]. In the few studies that evaluated the hormonal impact of probiotic supplementation, supplementation was associated with an increase in SHBG and a decrease in total testosterone levels [68].

A more recent meta-analysis and systematic review specifically studied the impact of prebiotics and symbiotic supplementation on the cardiometabolic parameters of women with PCOS, including 20 studies for review [69]. The researchers found high-quality evidence to support supplementation for reducing BMI and diastolic blood pressure without any improvement in systolic blood pressure [69]. There was moderate-quality evidence to suggest that supplementation benefits overall weight, the waist-to-height ratio, and triglycerides [69]. There was lower-quality evidence to support improvements in waist circumference, fat mass, fasting plasma glucose, fasting insulin, LDL, total cholesterol, high-sensitivity CRP, and total testosterone [69]. In the subgroup analysis, synbiotic supplementation, specifically, was associated with improvements in waist circumference, total cholesterol, triglycerides, and total testosterone [69]. In six studies, there was no significant difference in SHBG levels [69].

A recent RCT included patients with PCOS who were undergoing intensive lifestyle modifications, including a regimented low-carbohydrate and low-fat diet, calorie restriction, and 30 to 40 min of exercise per day, then randomized them into a synbiotic supplementation intervention group and a placebo group [70]. The intervention group consumed the synbiotic, SANPROBI Super Formula, which contains multiple strains of bifidobacterium and lactobacillus [70]. After 6 months, both groups had a significant decline in BMI and body fat compared to baseline, although no significant difference in decline was found between the two groups [70]. Both groups also showed a significant improvement in hirsutism [70]. The intervention group showed a statistically greater improvement in waist and high circumferences compared to the placebo [70]. Most profound was the impact of synbiotic supplementation on testosterone. One hundred percent of women in the intervention group had a decline in testosterone, compared to 5% of women in the placebo group. Furthermore, the average change in total testosterone was 40% in the synbiotic intervention group, compared to 5% in the placebo group [70]. The intervention group also showed significantly greater improvements in fasting insulin, insulin sensitivity index, total cholesterol, LDL, and triglycerides compared to the control [70]. This study highlights the impact of lifestyle interventions, with further improvements shown in metabolic and hormonal parameters with synbiotic supplementation.

These studies suggest that there is a potential benefit in probiotic supplementation as a complementary therapeutic option in the management of PCOS, used in addition to lifestyle modifications to improve insulin sensitivity, lipid profile, and hormonal and anthropomorphic parameters. However, evidence is mixed on the strength of the effect and variations in the efficacy of prebiotics, synbiotics, and probiotics.

Supplementation with vitamin D, antioxidant therapy, and probiotics offer effective adjuncts to the dietary management of PCOS and obesity to address the underlying role of chronic local inflammation, oxidative stress, and gut dysbiosis, thereby contributing to insulin resistance, hyperandrogenism, and metabolic dysfunction.

#### 5.1.3. Physical Activity

Similar to dietary considerations, the 2023 International Evidence-based Guideline for PCOS does not specify the ideal type or the intensity of exercise training for PCOS outcomes. Rather, the guidelines advise against a sedentary lifestyle and recommend a goal of 150 to 300 min of moderate-intensity activity or 75 to 150 min of vigorous-intensity aerobic activity per week, alongside strengthening exercises. For individuals with the goal of weight loss, then, a minimum of 250 min/week of moderate activity or 150 min of vigorous activity is recommended [5]. In a prospective baseline randomized control trial of women with PCOS, participants were assigned to either a control group with an unaltered lifestyle or to a 3-month structured exercise training program. The intervention training program was found to yield improvements in cardiopulmonary functional capacity (peak oxygen consumption and maximal workload) and fasting insulin, with reductions in BMI and CRP. However, there was no significant difference in hormonal parameters, including androgen levels, SHBG, LH, and FSH [6,71]. Aerobic workouts, in general, have been found to improve insulin sensitivity and cardiovascular health [2].

In a systematic review on the impact of exercise on hormonal parameters in women with PCOS, aerobic exercise was shown to have some positive effect on insulin sensitivity (fasting insulin or HOMA-IR), especially with higher-intensity exercise, although sex hormones were generally unchanged [72]. One study found a significant decrease in AMH with aerobic exercise; however, a separate study found no significant difference [72]. HIIT (high-intensity interval training) was also associated with a positive impact by lowering fasting insulin and HOMA-IR, with more studies also indicating a positive impact on sex hormones, with some reporting lower testosterone and increased SHBG [72]. In two non-randomized studies reviewing resistance exercise, there was a positive impact on decreased testosterone, although evidence is limited [72]. Similarly, there were two randomized controlled trials that reviewed yoga and indicated a benefit in terms of reducing testosterone [72,73,74]. Another systematic review and meta-analysis confirmed that aerobic exercise did not have a significant impact on hormonal parameters relating to PCOS, although they did not note a significant effect of aerobic exercise on HOMA-IR [75]. However, there is moderate evidence to suggest a significant impact of aerobic exercise on lowering BMI values [75].

Physical activity is important in PCOS due to its known benefits for cardiovascular and metabolic health. Furthermore, studies have shown that exercise can also improve depression scores in women with PCOS [76]. However, there is limited evidence to suggest the superiority of one type of exercise. There is currently limited evidence on the impact of physical activity on improving hormonal parameters. Type, duration, and intensity of exercise, therefore, should be personalized, based on other patient factors including age, functional status, cardiovascular risk, capacity, and comorbidities. This is an area that requires further research to guide any more specific recommendations.

#### 5.1.4. Behavioral Interventions

Women with PCOS have an increased risk of developing mental health disorders, including depression and anxiety, with an up to 8.1-fold increased risk of depression compared to women without PCOS, along with a prevalence of depression estimated at 36% and a prevalence of anxiety estimated at up to 76.7% [76,77]. Patients with PCOS and obesity were found to have significantly increased rates of depression compared to non-obese PCOS patients [77]. Similarly, women with both obesity and PCOS have statistically significantly higher rates of depression compared to women with obesity but without PCOS [77]. In patients with obesity and PCOS, the severity of their depression is also found to be higher [77]. Therefore, when caring for all patients with PCOS, and particularly for those who are also obese, it is important to address their mental health status and screen for depression, anxiety, and other related mental health disorders.

In a meta-analysis of women with PCOS, cognitive behavioral therapy was associated with improvements in anxiety and quality of life related to hirsutism [78]. In a randomized control trial of women with PCOS trying to conceive who had a BMI greater than 25 kg/m^2^, participants were randomized to either usual care as controls or an intervention group with a three-component lifestyle intervention—cognitive behavioral therapy, nutritional advice, and exercise [79]. After 12 months, the intervention group reported significantly greater weight loss than those receiving the usual care, with greater weight loss reported in patients who received additional support through the Short Message Service (SMS) from the psychologist to their phones after 3 months of the lifestyle program [79]. In the intervention group, 52.8% of women achieved a weight reduction greater than 5%, and 85.7% of women who received the SMS reached this goal, compared to 21.8% in the usual care group [79]. Therefore, it was concluded that the three-part behavioral intervention program led to significant weight loss in women with PCOS and obesity [79]. The intervention group was also found to show significant within-group improvements in ovulatory dysfunction and PCOM from baseline, with weight loss contributing positively to improvements in ovulatory dysfunction and hyperandrogenism [80]. Upon secondary analysis from this study, the intervention group also exhibited significant improvements in depression, self-esteem, and disordered eating compared to the control group. The improvement in depression was independent of weight loss and androgen level [81,82]. The results from this RCT support the use of an intentional and personalized intervention with dietary advice, physical activity, and mental health components for patients. Similarly, a smaller RCT found improvements in anxiety, depression, and quality of life with mindfulness stress management training [2,83].

### 5.2. Pharmacotherapy

#### 5.2.1. Metformin

Although not currently indicated for weight loss, metformin is recommended as a first-line therapy in addition to lifestyle modifications for patients with PCOS and a BMI of ≥25 kg/m^2^, due to improvements in anthropometric and metabolic outcomes [5]. Metformin is a biguanide that functions by activating the adenosine monophosphate-activated protein kinase (AMPK) pathway, which inhibits the hepatic production of glucose, reduces the oxidation of fatty acids, and increases the peripheral tissue uptake of glucose [6]. Metformin improves insulin sensitivity and fasting insulin levels, irrespective of BMI [6]. Furthermore, metformin has been demonstrated to lower the elevated serum AMH levels seen in women with PCOS [84].

In a systematic review of women with PCOS, metformin was associated with a larger reduction in BMI, HOMA-IR, and fasting glucose compared to the placebo [85]. In combination with a low glycemic index diet, there was a great improvement in the insulin sensitivity index, using the oral glucose tolerance test as a proxy, in women prescribed metformin [49]. In terms of AMH, metformin has been found to decrease AMH levels and normalize elevated levels, which likely contribute to this improved ovulatory success [12]. In studies, the decrease in AMH is delayed and has been found to take 3–6 months of treatment [12]. For ovulation, metformin has been found to improve ovulation rates when used as a co-treatment with clomiphene citrate compared to clomiphene or metformin monotherapy, and this trend was stable across the BMI subgroups [6]. Compared with the placebo, metformin was also found to significantly reduce total serum testosterone, although with a strong effect in women without obesity but with PCOS [6,86]. Furthermore, women without obesity had improved clinical pregnancy outcomes compared to women with obesity [6]. Despite its success in the preconception period to improve the PCOS phenotype in terms of hyperandrogenism, ovulatory function, and insulin sensitivity, metformin use in pregnancy has not been found to reduce the risk of gestational diabetes, hypertension of pregnancy, preeclampsia, macrosomia, or late spontaneous abortion [5,87].

The limitations of metformin therapy include adverse side effects limiting the tolerability of the medication, including bloating, abdominal pain, nausea/vomiting, diarrhea, and abnormal liver function tests [6].

#### 5.2.2. GLP-1 Receptor Agonists

The glucagon-like peptide is a natural hormone that is primarily synthesized in the small intestine and, in smaller quantities, in the brain and pancreas [88]. Glucagon-like peptides stimulate pancreatic insulin secretion, increase glucose transporters, inhibit glucagon release, and slow gastric emptying [33]. This results in glucose uptake, glycogen synthesis, and earlier satiety [33]. Receptors are present in the pancreas, the gastrointestinal tract, and the nervous system. GLP acts centrally on the signaling responsible for the homeostasis of energy balance to further contribute to weight loss [88]. GLP-1 receptor agonists are pharmacologically produced, and current formulations include liraglutide, semaglutide, tirzepatide, dulaglutide, exenatide, and lixisenatide [6,33]. Indications for medical management include weight loss, for type 2 diabetes patients with an established or high risk of atherosclerotic cardiac disease, or as a second-line treatment for type 2 diabetes, non-alcoholic steatohepatitis, and obstructive sleep apnea [33]. PCOS is currently not an indication for GLP-1 receptor agonist treatment.

In terms of weight loss, GLP-1 receptor agonists are highly effective. Semaglutide was found to yield a 14.9–17.9% weight loss after 68 weeks [89,90,91,92,93]. Tirzepatide was found to contribute to 22.9% of weight loss after 1 year, with a plateau for weight loss achieved at 3 to 6 months, although this was maintained with ongoing medical therapy [33,94,95]. In a meta-analysis evaluating pharmacologic therapies for the management of weight loss in women with PCOS and obesity, liraglutide was found to be more effective at weight loss and for reducing waist circumference, in comparison to metformin or a combination of metformin and liraglutide, which is likely due to the reduced dose of liraglutide in combination therapy [87]. GLP-1 receptor agonists have also been associated with the lowering of LDL cholesterol and triglycerides [88].

In terms of PCOS, GLP-1 receptor agonists have been found to reduce PCO morphology, decrease biochemical hyperandrogenism, and improve menstrual regularity and ovulatory function [33]. In a small study of 27 patients with PCOS and obesity who were unable to lose weight after lifestyle modifications, a 12-week treatment with weekly semaglutide administration was associated with significant weight loss [96]. Eighty percent of the study population achieved at least a 5% weight reduction at 3 months, and 80% of these responders reported the normalization of their menstrual cycles [96]. GLP-1 therapy was also associated with reduced fasting insulin levels and improved HOMA-IR, independent of weight loss [96]. In a small retrospective study comparing patients who were overweight or obese with PCOS versus controls who did not have PCOS, there was no significant difference in the weight loss achieved between patients with PCOS and the control group, with improved metabolic parameters in both groups [87]. However, in patients with PCOS who had over a 5% weight reduction, they were more likely to be white in terms of race, have a lower baseline BMI, and meet all three Rotterdam criteria [87]. In a meta-analysis of GLP therapy in the general population, GLP-1 receptor agonist therapy was found to improve depressive symptoms compared with controls [97]. Liraglutide was also associated with an improvement in psychological health, as recorded on the World Health Organization Quality of Life brief version questionnaire, but was not associated with lowering the percentage of individuals who screened positive for depressive symptoms [76,98].

As metformin has been part of the front-line therapy for PCOS following lifestyle modifications, due to improvements in insulin sensitivity, there are multiple studies comparing the efficacy of GLP-1 therapy compared to metformin. In an RCT of women with PCOS who were overweight, which compared single and combined treatments of exenatide and metformin, a combination therapy was found to significantly improve ovulation to 86%, compared to 50% in exenatide monotherapy and 29% in metformin monotherapy. The combined therapy was also associated with decreased total cholesterol, triglycerides, and improved menstrual regularity [99]. Similarly, in another small, randomized, prospective study comparing metformin monotherapy to combination metformin and semaglutide in patients with PCOS with infertility, the combination treatment was found to yield a greater recovery of menstrual regularity, higher pregnancy rates during the follow-up period after semaglutide discontinuation, a significant reduction in CRP, a significant reduction in HDL and LDL, a greater reduction in testosterone and increase in SHBG, and a reduction in BMI in comparison to metformin monotherapy [100]. However, both treatment groups showed significant reductions in hemoglobin A1c and HOMA-IR compared to baseline [100]. In a small open-label randomized study of 28 women with PCOS, infertility, and obesity, 12 weeks of preconception treatment of liraglutide with metformin was found to be more beneficial than metformin monotherapy for improving the IVF pregnancy rate per embryo transfer and spontaneous pregnancy rates [33,101]. In a meta-analysis comparing GLP-1 receptor agonists versus metformin in PCOS, liraglutide was associated with decreased total testosterone levels and BMI. Compared to metformin, GLP-1 receptor agonist therapy was found to be more effective for improving insulin sensitivity and reducing BMI in women with PCOS [6,102].

Despite positive data regarding the use of GLP-1 receptor agonist therapy for the treatment of individuals with PCOS and obesity, limitations do exist. In the United States, GLP-1 receptor agonists are costly medications with a net cost of USD 700 to 800 per month, accounting for drug discounts [103]. Insurance coverage has become more inclusive, with expanded FDA indications. However, in the United States, Medicare only provides coverage for specific indications. For example, semaglutide is covered for diabetes and cardiovascular disease, while tirzepatide is covered for sleep apnea [103]. Private insurers frequently require prior authorization for coverage of these medications and for inclusion in weight management programs [104]. Currently, in the United States, there is no insurance coverage for the use of these medications for reproductive indications or PCOS. Overall, the relevance of these insurance criteria varies from country to country, depending on the healthcare delivery system.

GLP-1 therapy is associated with severe adverse effects, including nausea, diarrhea, constipation, and abdominal pain. Furthermore, the teratogenicity potential of these medications is unclear; therefore, preconception use is limited, and its use in pregnancy is currently not recommended. Animal studies were associated with possible embryofetal mortality, structural abnormalities, and growth alterations [33]. Currently, there are not sufficient human studies to ensure its safety of use in early pregnancy [33], although retrospective data on pregnancies conceived along with inadvertent early exposure to GLP-1 therapy have been reassuring [33,105]. Given this unclear evidence, the manufacturers of semaglutide and tirzepatide currently recommend discontinuing medical therapy 2 months prior to attempting conception [33].

#### 5.2.3. Thiazolidinediones

Thiazolidinediones are a class of agents used for the control of type 2 diabetes, and are PPAR-γ agonists. Their mechanism of action involves increasing the number of intracellular peroxisomes to aid in the breakdown of toxins [3]. PPAR-γ also aids in the uptake of insulin-dependent glucose while decreasing the hepatic glucose output, thereby improving insulin sensitivity [3]. Thiazolidinediones have fallen out of favor, given concerns regarding weight gain, peripheral edema, and worsening cardiovascular risk in patients with underlying risk; therefore, they are not first-line agents and are more often second- or third-line agents [3,58]. Currently available formulations include pioglitazone, as rosiglitazone is rarely used due to data supporting a potential increased risk of cardiovascular events [106].

With regard to PCOS, thiazolidinediones have been shown to improve glycemic and metabolic parameters by reducing HOMA-IR, fasting plasma glucose levels, triglycerides, and LDL cholesterol and increasing HDL cholesterol [3]. In a meta-analysis of the different insulin sensitizers in individuals with PCOS, thiazolidinediones were superior to metformin in terms of increasing HDL cholesterol, reducing LDL cholesterol, and decreasing fasting plasma glucose and triglycerides. Thiazolidinediones were also associated with fewer of the gastrointestinal side effects that limit metformin’s tolerability [4]. Metformin and thiazolidinediones, when used in combination, were associated with lower triglyceride levels compared to monotherapy [4]. Similarly, combination therapy was also more efficacious than the use of metformin alone for improving menstrual regularity [4]. Based on this evidence, thiazolidinediones may function as an effective adjunct in appropriate patients with persistent metabolic and ovulatory dysfunction. However, in patients with obesity, the elevated cardiovascular risk and potential weight gain make this class of medications less favorable for the management of PCOS.

#### 5.2.4. Orlistat

Orlistat is an FDA (Food and Drug Administration)-approved weight loss medication that functions by inhibiting gastric and pancreatic lipases, resulting in the reduced absorption of fat and increased excretion, along with a reduced caloric intake [33]. Orlistat has been effective for producing and sustaining weight loss and a reduction in cardiovascular risk factors [107]. Orlistat has also been found to improve blood pressure, insulin sensitivity, and lipid profile [33].

In the case of PCOS, orlistat has been associated with improved insulin sensitivity and hyperandrogenism [9]. In a systematic review and meta-analysis examining orlistat use in women with PCOS, orlistat was found to be associated in most studies with a significant improvement in insulin sensitivity with a decrease in HOMA IR and/or insulin levels, as well as a significant reduction in testosterone levels [107]. Of the few studies that evaluated menstrual cycles, there was no improvement in regularity reported with orlistat administration [107]. Both metformin and orlistat were found to yield improved ovulation rates compared to the control group, and there was no significant difference in ovulation rates between metformin and orlistat [107]. Similarly, orlistat and metformin both yielded a similar reduction in BMI, HOMA-IR, testosterone, and insulin [107]. Orlistat has not been found to improve clinical pregnancy, live births, or conception, despite significant associated weight loss [9,33].

Adverse effects attributed to orlistat include oily stools, flatulence, and fat-absorbed vitamin deficiencies (vitamins A, D, E, K) [107]. Orlistat is contraindicated in patients with underlying risk factors for malabsorption and cholestasis [107]. Its impact on vitamin D absorption has been hypothesized as a potential explanation for an improvement in weight loss that was not associated with reproductive outcomes. As mentioned previously, vitamin D deficiency has been implicated as a plausible pathway exacerbating the PCOS phenotype and adverse reproductive outcomes [33]. Similar to other weight loss medications, orlistat is recommended for discontinuation prior to conception [108].

#### 5.2.5. Naltrexone-Bupropion

Naltrexone-bupropion is a combination weight loss regimen [33]. Naltrexone blocks μ-opioid receptors in the brain, which inhibits the reward centers in the hypothalamus linked to food and acts to modulate hunger signals and suppress the drive to consume food [33]. Bupropion, used for selected mood disorders, inhibits the reuptake of dopamine and norepinephrine to improve mood and energy, and also reduces appetite [33]. This combination of medications acts synergistically for weight loss [33]. Naltrexone-bupropion has been found to lead to significant weight loss, alongside a reduction in triglycerides and enhanced glycemic control with T2DM (type 2 diabetes mellitus) [33,109].

Currently, there are limited studies regarding the use of naltrexone-bupropion specifically for the management of PCOS. However, PCOS is associated with an increased risk of both mood disorders and eating disorders. Specifically, binge-eating disorder is one of the most prevalent eating disorders in individuals with PCOS, with this risk being increased in individuals with comorbid obesity [110]. Approximately 33% of individuals with PCOS have disordered eating patterns, and 6% have eating patterns that are consistent with binge-eating disorders [110]. Naltrexone-bupropion has been found to have a positive effect in terms of weight loss and on binge-eating disorder in individuals, although remission was found to be limited after medication discontinuation [110].

Use of naltrexone-bupropion is contraindicated in those patients with chronic opioid use disorder or who are in withdrawal, with hypertension, seizure disorder, bulimia or anorexia, benzodiazepines, barbiturates, and monoamine oxidase (MAO) inhibitors, due to its mechanism as an opiate receptor inhibitor and with bupropion lowering the seizure threshold [33,111]. The most frequent adverse effects include headaches, constipation, vomiting, dry mouth, dizziness, and a transient increase in blood pressure [33,111]. In animal studies, high doses have been associated with fetal loss and developmental anomalies [33,111].

#### 5.2.6. Phentermine-Topiramate

Phentermine-topiramate is a combination pharmacotherapy that has been approved for weight loss [33]. Phentermine is responsible for stimulating the release of norepinephrine to increase energy expenditure and satiety [33]. Topiramate modulates the GABA and AMPA receptors, thereby reducing cravings [33]. Both medications improve satiety by their respective pathways. The combination therapy was found to be more effective for weight loss than when either is administered as a monotherapy [33,34]. In a randomized, placebo-controlled phase 3 trial, participants with a BMI of 27–45 kg/m^2^ and two more comorbidities were randomly assigned to groups receiving a placebo versus a low dose of 7.6 mg phentermine and 46 mg topiramate or a high dose of 15 mg phentermine and 92 mg topiramate. After 56 weeks of treatment, patients in both the lower-dose and higher-dose groups had significantly greater weight loss compared to the placebo group (−8.1 kg and −10.2 kg, respectively) [112].

In a 24-week RCT of individuals with PCOS and obesity (BMI 30–45 kg/m^2^), comparing exenatide (GLP-1 receptor agonist), combination exenatide (EQW), and dapagliflozin, combination dapagliflozin (DAPA) and metformin, and phentermine and topiramate (PHEN/TPM), the OGTT (oral glucose tolerance test) and HOMA-IRA values were improved in all treatment groups. Both EQW/DAPA and PHEM/TPM were more effective in reducing body weight, total body fat by DXA, and waist circumference. Total testosterone, free androgen index, and SHBG levels were significantly improved in all treatment groups. EQW/DAPA yielded a significant reduction in triglycerides compared to PHEN/TPM [113]. Furthermore, short courses of phentermine have been associated with improved conception following its discontinuation [114]. In a small retrospective study evaluating the use of a 3-month treatment of phentermine in the preconception period in individuals with obesity and infertility, the mean percentage weight reduction was 5.3%, the pregnancy rate was 60%, and the live birth rate was 49% [114]. There was no difference in live birth rate or pregnancy rate in individuals who lost more than or less than 5% of their starting body weight [114].

Adverse events related to phentermine-topiramate include dry mouth, paresthesia, constipation, insomnia, dizziness, dysgeusia, anxiety, and depression [112]. Phentermine is currently only approved for short-term use [34]. Topiramate has been associated with teratogenicity with fetal malformation and growth defects, and its impact on GABA receptors could theoretically impair ovulation and oocyte quality [33]. Therefore, patients on this medication need to be counseled regarding initiating effective contraception.

In summary, there are many effective medications on the market that can lead to significant weight loss, with many also conferring benefits in terms of improving insulin sensitivity and metabolic parameters. However, their use for reproductive outcomes is limited as most of these medications, save for metformin, are contraindicated while actively trying to conceive, due to a lack of data on early fetal development. These medications can benefit those individuals seeking weight loss prior to trying to conceive or those who are not interested in fertility and desire to improve other comorbidities and the risks associated with PCOS and obesity. Medication selection should be personalized, based on the patient’s underlying goals, comorbidities, and side effect profile.

### 5.3. Surgical Management

The American Society for Metabolic and Bariatric Surgery and the International Federation for the Surgery of Obesity and Metabolic Disorders recommend metabolic and bariatric surgery (MBS) for individuals with a BMI of ≥35 kg/m^2^, regardless of comorbidities, and for those individuals with a BMI of ≥30 kg/m^2^ and with T2DM. MBS may also be considered for individuals with a BMI of 30–35 kg/m^2^ who do not achieve weight loss or co-morbidity improvement after nonsurgical interventions [115]. Metabolic and bariatric surgery functions through restriction, malabsorption, and changes in gut signaling. Bariatric surgery reduces gastric capacity and caloric intake. It also leads to alterations in gut hormone signaling. GLP-1 and peptide YY levels are increased by stimulating satiety centrally while decreasing the ghrelin levels to reduce hunger signals [9]. Currently, the most common procedures include the Roux-en-Y gastric bypass and sleeve gastrectomy. Less common procedures include adjustable gastric banding, biliopancreatic diversion with a duodenal switch, and one anastomosis gastric bypass [115]. Bariatric surgery can result in a 60–70% loss of excess weight within 12 months, and, over 5 years, post-surgery patients are able to maintain 50% of their excess weight loss [33,34].

Specifically in the PCOS population, individuals showed more significant and prolonged weight loss with bariatric surgery compared to low-calorie diets [9]. In meta-analysis, bariatric surgery has been associated with improvements in total testosterone levels, fasting blood glucose, and insulin, along with significant reductions in triglycerides, self-reported acne, and hirsutism [9]. Several studies have also validated the restoration of normal menstruation [9,116,117,118]. In another meta-analysis including women with obesity who underwent bariatric surgery, 36% of patients had a diagnosis of PCOS prior to surgery. Following surgery, weight loss was associated with a 96% resolution rate of PCOS, resolution of hirsutism, a 95% resolution of menstrual dysfunction, and an improvement in PCO morphology [6,119]. In a prospective, multicenter, non-randomized trial in premenopausal women, 122 individuals with severe obesity reported menstrual irregularities; of these, 60 had a diagnosis of PCOS and 62 did not [120]. The control group in this study included women who declined bariatric surgery or weight reduction medications [120]. The intermenstrual interval was shortened towards a normal length in both PCOS and non-PCOS women with previously abnormal menstrual cycles who underwent gastric sleeve resection at both 6 and 12 months postoperatively [120]. Ovulatory function was achieved in 63.6% of subjects with PCOS and 45% of subjects without PCOS at 6 months postoperatively, both percentages being significantly higher than in the control group [120]. Additionally, the percentage rose significantly in the PCOS group at 12 and 15 months [120].

Bariatric surgery has also been evaluated in terms of its impact on reproductive and fertility outcomes. In several prospective cohort studies, bariatric surgery was associated with significantly improved pregnancy rates in nulliparous women with obesity [9,33]. In a small study of 216 patients, pregnancy and live birth rates in patients with PCOS following bariatric surgery were 95% and 68%, respectively [6,121]. In a retrospective cohort study of all patients with prior bariatric surgery who subsequently underwent IVF treatments, individuals who regained less than 3 points of their BMI were approximately twice as likely to have a clinical pregnancy during a follow-up period of 36 months, after adjusting for age and current BMI, which were not significantly associated with clinical pregnancy [122]. This population was also three times more likely to have a live birth during this follow-up period, and obesity was not associated with live birth [122]. This study found that even a relatively small regain of weight of >3 units of BMI could have a negative impact on clinical pregnancy and live birth rates following bariatric surgery [122]. Due to rapid weight loss, there was a theoretical concern regarding an increased risk of fetal growth restriction, although more recent evidence has been reassuring, with the benefits of weight loss by reducing obesity-related obstetric complications (gestational diabetes, preeclampsia, and pregnancy-related hypertension) outweighing the risks [6,121,123,124].

Current guidance recommends waiting one to two years after bariatric surgery (1 year after a sleeve gastrectomy or Roux-en-Y gastric bypass and 2 years after adjustable gastric banding) before conceiving to allow for the stabilization of weight [125]. In a large retrospective cohort study comparing perinatal complications between women with a history of bariatric surgery and women without a history of bariatric surgery, infants from mothers who were less than 2 years postoperative from surgery had a significantly greater relative risk of prematurity, NICU (neonatal intensive care unit) admission, and SGA (small for gestational age) status compared to infants from mothers who were longer than 2 years postoperative from bariatric surgery [126]. In general, infants from mothers with a history of bariatric surgery had a higher relative risk of prematurity, NICU admission, SGA status, and low Apgar score compared to infants from mothers without a history in this population-based random sample, which was matched by delivery year, with the relative risk adjusted based on multiple maternal covariates, including BMI, hypertension, and diabetes [126].

Overall, bariatric surgery for individuals with comorbid PCOS and obesity has been found to yield significant benefits in terms of weight loss, menstrual irregularities, and insulin resistance. Some evidence suggests likely fertility and reproductive benefits as well, although there has been evidence to suggest a slight increase in perinatal complications. However, these risks should be weighed against the obstetric risks associated with obesity. Additional limitations of bariatric surgery include limited access to services, the ability to undergo preoperative clearance and follow-ups with a multidisciplinary team, and comorbidities incurring additional risks associated with surgery and anesthesia.

## 6. The Future of Personalized Medicine

Given the advancements in burgeoning pharmacotherapies and minimally invasive bariatric surgery, there are now multiple effective weight loss options for individuals with PCOS to improve their reproductive health and reduce cardiovascular and metabolic risk. Table 1 summarizes the efficacy of the different dietary, pharmacotherapy, and surgical options available to manage co-morbid PCOS and obesity and to improve metabolic, cardiovascular, and reproductive outcomes. For example, different dietary regimens can most benefit those patients pending their underlying comorbidities and genetic profile, including hypertension, prediabetes or diabetes, and a history of eating disorders. Patients can also benefit from supplementation if they are deficient in vitamin D. However, personalized diets are necessary to complement the patients’ own food and cultural preferences. Pharmacotherapy offers an effective adjunct with lifestyle modifications, with the risks and benefits of each medication class pending patients’ underlying health risks, the desired timeline of weight loss, and fertility considerations. Lastly, bariatric surgery has been proven to be a highly effective and successful weight loss option to reduce cardiometabolic risk factors. However, surgery requires a preoperative commitment to a nutritional plan, the assessment of preoperative risk factors, the ability to maintain a restricted diet in the long term, and the time and resources for recovery and follow-ups. Therefore, given varying preconception concerns, personal goals, and priorities, with Figure 1 providing examples, adverse effects, and time and resource constraints, a personalized approach is required to develop effective, patient-centered treatment interventions that are both feasible and sustainable in the long term.

The Michigan Interdisciplinary Clinic for Obesity and Reproduction is a university-based multidisciplinary clinic designed for individuals who are overweight/obese and desirous of current or future fertility, with a reproductive disorder [128]. This clinic has developed a personalized program for weight loss, with a large team equipped with a nutritionist, social worker, maternal fetal medicine specialist, obesity medicine specialist, and reproductive endocrinologist who function together to develop weight loss plans. The plans incorporate a meal plan developed with a nutritionist, counseling for bariatric surgery in appropriate candidates, and anti-obesity medications [128]. In a retrospective cohort analysis of patients enrolled in this clinic, 63% of the population were willing to postpone pregnancy for at least 3 months to optimize weight loss prior to trying to conceive [128]. The patients who were willing to defer had a higher baseline AMH and were less likely to have diabetes or anxiety compared to those patients who did not agree to defer. Patients who were willing to defer fertility were more willing to achieve more than 5% of weight loss after a 6-month period. Significantly, patients who were able to achieve more than 5% of weight loss at 5 months were more likely to achieve pregnancy within the first 6 months of trying to conceive and beyond 6 months, without a difference in spontaneous abortion rates. Willingness to defer pregnancy was the only predictor of achieving more than 5% of weight loss at 6 months [128]. This clinic has approached weight loss and the management of obesity with a personalized approach, leading to successful outcomes for those patients committed to achieving their goals—in this example, fertility. This clinic exemplifies a personalized approach to developing sustainable plans addressing social, medical, and psychiatric barriers.

Furthermore, despite the effectiveness of treatment options, there is still heterogeneity in terms of responders versus non-responders, based on the different options. Therefore, personalized responses are necessary to identify interventions that match each individual’s biopsychosocial and genetic profile. A recent 12-week pilot study was performed to evaluate the utility of a 12-week personalized behavioral weight loss program in comparison to behavioral weight loss programs that follow standard diet and activity recommendations for all participants [129]. In this study, both groups had self-monitoring devices/sensors to track their progress (a Fitbit and a Bluetooth scale), as well as regular weekly check-ins to monitor progress. The personalized behavior weight loss intervention group had their caloric goals established, based on their personal energy expenditure, along with fitness goals that were set based on the individual’s progress, and were given a continuous glucose monitor to help individuals experience in real time the effect of diet on glycemic control. The standard behavioral weight loss group had caloric goals that were set based on their starting weight and a standardized increased progression for physical activity with pre-set intervals. In this study, the personalized behavioral weight loss group reported significantly greater weight loss and also had a greater percentage of participants who had achieved a >5% weight loss at 3 months [129]. This study represents the value of developing weight loss programs centered around an individual’s own baseline lifestyle and goals.

## 7. Conclusions

Polycystic ovary syndrome is a common and complex endocrinopathy with serious reproductive, metabolic, and cardiovascular health implications if it is not appropriately managed and treated. PCOS increases the risk of obesity, and, together, obesity and PCOS exacerbate cardiovascular health risks and can result in more extreme phenotypic presentations of PCOS. Weight loss is the backbone of the management of PCOS in individuals who are overweight or obese. Weight loss typically requires the patient’s significant motivation to create sustainable change. Anti-obesity medications and bariatric surgery have served as effective adjuncts when lifestyle modifications are not sufficient to reduce the risks associated with obesity-related comorbidities. There are innumerable patient factors that limit the efficacy of standardized weight loss plans, including food allergies, medical comorbidities, limited access to health food and medical technologies, time constraints regarding commitment to a rigorous program, and genetic variations in the pharmacokinetic metabolism of medications. Therefore, when approaching the reduction of obesity in PCOS, a personalized approach should be considered to assist in developing a plan that is effective, safe, achievable, and sustainable by being aligned with the individual patient’s own personal goals and aspirations regarding their health.

## Figures and Tables

**Table 1:** Summary of the various interventions and their impacts on anthropomorphic, metabolic, androgenic, and reproductive outcomes in individuals with PCOS.

Intervention	Description	Anthropomorphic Parameters	Metabolic Parameters	Androgenic and Reproductive Parameters	Adverse Effects/Risks
Diet					
Low glycemic index [49,50,51]	Foods with a low, steady rise in blood sugar levels (i.e., whole grains, vegetables, fruits)	- Reduces BMI - Reduces waist circumference	- Decreases HOMA-IR, fasting glucose, fasting insulin levels - Decreases total cholesterol	- Improves ovulatory cycles and menstrual regularity	None
Ketogenic [2,3,52,53]	Low in carbohydrate, adequate protein, high in fat (regular ketogenic diet) or low-fat content (very low-calorie ketogenic diet or very low-energy ketogenic therapy)	- Reduces BMI - Reduces fat mass and waist circumference	- Decreases total cholesterol and triglycerides - Decreases hemoglobin A1c and LDL with VLEKT - Decreases serum glucose, HOMA-IR	- Lowers androgen levels - Decreases LH/FSH - Increases SHBG	Electrolyte imbalances, dehydration, nutritional deficiencies
DASH [51,54,55]	Balanced diet with lean meats, vegetables and fruits, low glycemic index carbohydrates, micronutrients, sodium restriction	- Reduces BMI	- Decreases HOMA-IR, fasting insulin, fasting glucose	- Not found to significantly reduce total testosterone levels	None
Supplementation					
Vitamin D [3,6,12,17,18,19,57,58,127]	Vitamin D supplementation		- Decreases CRP and malondialdehyde - Improvement in glucose homeostasis	- Decreases total testosterone and androstenedione - Decreases in serum AMH	None
Anti-inflammatory [2]	Rich in fruits, vegetables, omega-3 fatty acids, whole grains		- Decreases cholesterol, triglycerides - Improves insulin sensitivity - Improves serum adiponectin	- Decreases LH/FSH - Improves menstrual regularity	None
Antioxidant [3,62,63,64,65]	Coenzyme Q10, vitamin E, resveratrol	- No significant impact on BMI	- Decreases CRP, malondialdehyde - DReduces fasting glucose and insulin, HOMA-IR - Decreases total cholesterol, triglycerides	- Decreases total testosterone, DHEA-S - Mixed evidence on decreasing hirsutism - Decreases in prolactin	None
Microbiome [66,67,68,69,70]	Probiotic or synbiotic supplementation, micro-biome rich foods include high fiber fermented foods, grains, and seeds	- Mixed evidence on improving BMI, waist circumference	- Decreases triglycerides, LDL - Decreases insulin levels - Decreases in HOMA-IR	- Decreases total testosterone - Increases SHBG	None
Exercise					
Aerobic [2,72,75]	Continuous use of large muscle groups, with increased heart rate	- Reduces BMI	- Improves insulin sensitivity (fasting insulin, HOMA-IR	- Mixed evidence on decreasing serum AMH - Sex hormones unchanged	Risk of injury, caution in certain cardiac populations, orthopedic restrictions
High-Intensity Interval Training [72]	High-intensity intervals with low-intensity rest intervals in between		- Decreases fasting insulin and HOMA-IR	- Decreases lower testosterone, increased SHBG	Risk of injury, caution in certain cardiac populations, orthopedic restrictions
Yoga [72]	Controlled breathing exercises with a sequence of held positions			- Decreases testosterone	Risk of injury, caution in certain cardiac populations, orthopedic restrictions
Pharmacotherapy					
Metformin [5,6,12,49,85]	Activates the APMK pathway, inhibits the hepatic production of glucose, and increases peripheral tissue uptake of glucose	- Reduces BMI - Reduces weight	- Decreases HOMA-IR and fasting glucose	- Decreases AMH levels - Improves ovulatory function with clomiphene citrate - Decreases total testosterone levels	Bloating, abdominal pain, nausea/vomiting, diarrhea, abnormal liver function tests
GLP-1 receptor agonist [6,33,76,87,88,96,101,102,113]	Stimulates pancreatic insulin secretion, inhibits glucagon release, slow stomach emptying	- Reduces BMI - Reduces weight	- Decreased fasting insulin levels and HOMA-IR - Decreased total cholesterol and triglycerides	- Increases menstrual regularity - Decreases total testosterone	Nausea, diarrhea, constipation, abdominal pain, potential teratogenicity
Thiazolidinediones [3,4,106]	PPAR-ᵧ agonist increases uptake of insulin-dependent glucose and decreases hepatic glucose production		- Decreases HOMA-IR, fasting plasma glucose levels - Decreases triglycerides, LDL cholesterol, increased HDL cholesterol	- In combination with metformin, improves menstrual regularity	Weight gain, increased risk of cardiovascular events, peripheral edema
Orlistat [9,33,107]	Inhibits gastric and pancreatic lipases	- Reduces BMI - Reduces weight	- Decreases HOMA-IR, insulin levels	- Decreases testosterone levels - No significant improvement in menstrual regularity - Improvement in ovulatory rates - Does not improve clinical pregnancy, live birth rates or conception	Oily stools, flatulence, fat-absorbed vitamin deficiencies; contraindicated with underlying risk factors for malabsorption and cholestasis
Naltrexone-bupropion [33,110]	Naltrexone blocks µ-opioid receptors, bupropion inhibits reuptake of dopamine and norepinephrine	- Reduces weight	- Decreases triglycerides		Headache, constipation, vomiting, dry mouth, dizziness, transient increase in blood pressure, potential teratogenicity. Contraindicated with chronic opioid use disorder or withdrawal, hypertension, seizure disorder, bulimia or anorexia, medications that lower the seizure threshold
Phentermine-topiramate [33,34,112,113,114]	Phentermine stimulates the release of norepinephrine, topiramate modulates GABA and AMPA receptors	- Reduces weight - Reduces total body fat and waist circumference	- Decreases triglycerides	- Decreases total testosterone, free androgen index, increases SHBG - Phentermine is associated with improved conception following discontinuation	Dry mouth, paresthesia, constipation, insomnia, dizziness, dysgeusia, anxiety, and depression
Surgery					
Gastric sleeve, Roux-en-Y gastric bypass, adjustable gastric banding [6,9,33,34,115,116,117,118,119,121,122,125,126]	Reduces gastric capacity and calorie intake, increases satiety via central signaling	- Reduces weight - Reduces BMI	- Reduces fasting glucose and insulin	- Decreases total testosterone levels - Decreases hirsutism and acne - Restores menstruation - Improves PCO morphology - Improves rate of ovulation - Improves clinical pregnancy rate and live birth rate with IVF	- Malabsorption, dumbing syndrome, surgical risks, anesthesia risks, potential increased risk of perinatal complications

Abbreviations: BMI (body mass index), HOMA-IR (homeostatic model assessment for insulin resistance), LDL (low-density lipoprotein), VLEKT (very-low-energy ketogenic therapy), LH (luteinizing hormone), FSH (follicle-stimulating hormone), SHBG (sex hormone-binding globulin), CRP (C-reactive protein), AMH (anti-Müllerian hormone), DHEA-S (dehydroepiandrosterone sulfate), AMPK (adenosine monophosphate-activated protein kinase), GLP (glucagon-like peptide), PPAR (peroxisome proliferator-activated receptor), HDL (high-density lipoprotein), GABA (gamma-aminobutyric acid), AMPA (alpha-amino-3-hydroxy-5-methyl-4-isoxazolepropionic acid), and PCO (polycystic ovary).

## Data Availability

No new data were created or analyzed in this study. Data sharing is not applicable to this article.
